# Chronic hyperglycemia regulates microglia polarization through ERK5

**DOI:** 10.18632/aging.101770

**Published:** 2019-01-26

**Authors:** Congde Chen, Suichun Wu, Zipu Hong, Xiaoming Chen, Xiaoou Shan, Shane Fischbach, Xiangwei Xiao

**Affiliations:** 1 Department of Pediatric Surgery, The Second Affiliated Hospital and Yuying Children’s Hospital of Wenzhou Medical University, Wenzhou 325000, China; 2 Reproductive Medicine Centre, The First Affiliated Hospital of Wenzhou Medical University, Wenzhou 325000, China; 3 Department of Pediatric Surgery, The First Affiliated Hospital of Wenzhou Medical University, Wenzhou 325000, China; 4 Department of Pediatric Endocrinology and Metabolism, The Second Affiliated Hospital and Yuying Children's Hospital of Wenzhou Medical University , Wenzhou 325000, China; 5 Department of Surgery, Children’s Hospital of Pittsburgh, University of Pittsburgh School of Medicine, Pittsburgh, PA 15224, USA

**Keywords:** extracellular-signal-regulated kinase 5 (ERK5), diabetes, Alzheimer’s disease (AD), microglia, polarization

## Abstract

Diabetic patients are prone to developing Alzheimer’s disease (AD), in which microglia play a critical role. However, the direct effect of high glucose (HG) on microglia and the role of extracellular-signal-regulated kinase 5 (ERK5) signaling in this interaction have not been examined before. Here, these questions were addressed in microglia cultured in HG versus normal glucose (NG) conditions. Initially, HG induced microglial differentiation into the M2a phenotype with concomitant ERK5 activation. However, longer exposure to HG further induced differentiation of microglia into the M2b-like phenotype, followed by the M1-like subtype, concomitant with a gradual loss of ERK5 activation. BIX021895, a specific inhibitor of ERK5 activation, prevented M2a- differentiation of microglia, but induced earlier M2b-like polarization followed by M1-like polarization. Transfection of microglia with a sustained activated form of MEK5 (MEK5DD) prolonged the duration of the M2a phenotype, and prevented later differentiation into the M2b/M1 subtype. Conditioned media from the M2a-polarized microglia reduced neuronal cell apoptosis in hypoxic condition, while media from M2b-like or M1-like microglia enhanced apoptosis. Together, our data suggest that chronic hyperglycemia may induce a gradual alteration of microglia polarization into an increasingly proinflammatory subtype, which could be suppressed by sustained activation of ERK5 signaling.

## Introduction

Alzheimer’s disease (AD), a common disease of the aging brain, is characterized by progressive loss of learning potential and memory [1]. During disease progression, proteostasis of amyloid-beta peptide aggregates (Aβ) and tau protein is gradually altered, resulting in the formation of senile plaques followed by neurofibrillary tangles (NFTs), two key pathological features of AD [2].

Diabetes is a prevalent metabolic disease that affects hundreds of millions of people worldwide [3]. Diabetic patients suffer from the loss of metabolic control of blood glucose, resulting from either reduced insulin production and secretion, or from development of insensitivity among insulin-responsive effector cells, or both. Diabetes has 2 major subtypes, type 1 diabetes (T1D) and type 2 diabetes (T2D) [4]. While T1D is characterized by immunological destruction of the insulin-producing beta cells [4], T2D is initiated by the loss of insulin sensitivity but is commonly followed by loss of functional beta cells [3].

Interestingly, recent evidence has revealed a higher risk of developing AD among T2D patients [5]. Mechanistically, this may be attributable to the chronic inflammatory environment in the diabetic brain, which impairs neuronal insulin signaling, synapse functionality and neuronal cell health [6,7]. However, the exact molecular mechanisms are still under exploration.

Microglia are the resident phagocytes of the central nervous system. Microglia are derived from infiltrated yolk sac progenitors during early embryonic development, and are maintained exclusively by self-proliferation in normal conditions, whereas they are partially maintained by circulating monocytes in disease conditions [8]. There is a diverse distribution of microglia in the adult brain: while in some regions microglia comprise as little as 0.5% of total brain cells, in other regions the percentage can be as high as 16.6% [9]. As a specific type of macrophage in the brain, microglia share a lot of features with macrophages and can be classified into several subtypes, including M1, M2a, M2b and M2c [10]. M1 microglia are associated with proinflammatory factors and cytokines, and exhibit significant expression of IL-6, TNF-α, IL-12, phagocytic oxidase like iNOS and MHC-II [10]. M2a is the typical M2, and has a strong anti-inflammatory signature, expressing IL-10, CD206, arginase 1 (Arg-1) and Chitinase-3-like-3 (in humans, and Ym1 in mice) [10]. M2b is a subtype between M1 and M2a, characterized by compromised levels of Arg-1, CD206, expression of the proinflammatory cytokines IL-12, IL-6, TNF-α, and low levels of iNOS [10]. M2c is an M2 subtype with high TGF-β and VEGF-A levels, and is associated with angiogenesis and immunosuppression [10]. These microglia subtypes can dynamically differentiate into each other, a process called polarization [11]. Since microglia have important functions in non-autonomous clearance of protein aggregates and in regulation of inflammation, they play critical roles in aging and neurodegeneration [11].

We have previously shown that macrophages and their polarization are essential for pancreatic beta cell growth and regeneration [12,13]. In the current study, we detected a direct effect of high glucose on microglia polarization, which is associated with pathological changes in AD. Importantly, we have previously shown that extracellular-signal-regulated kinase 5 (ERK5) is required for proper gestational pancreatic beta cell proliferation [14]. Here, we found that ERK5 signaling appeared to be required for a M2a polarization of microglia in response to high glucose. These data suggest a previously unacknowledged effect of chronic hyperglycemia on microglia polarization with implications for the development of AD.

## Results

### High glucose alters microglia polarization with time

**Figure 1:**
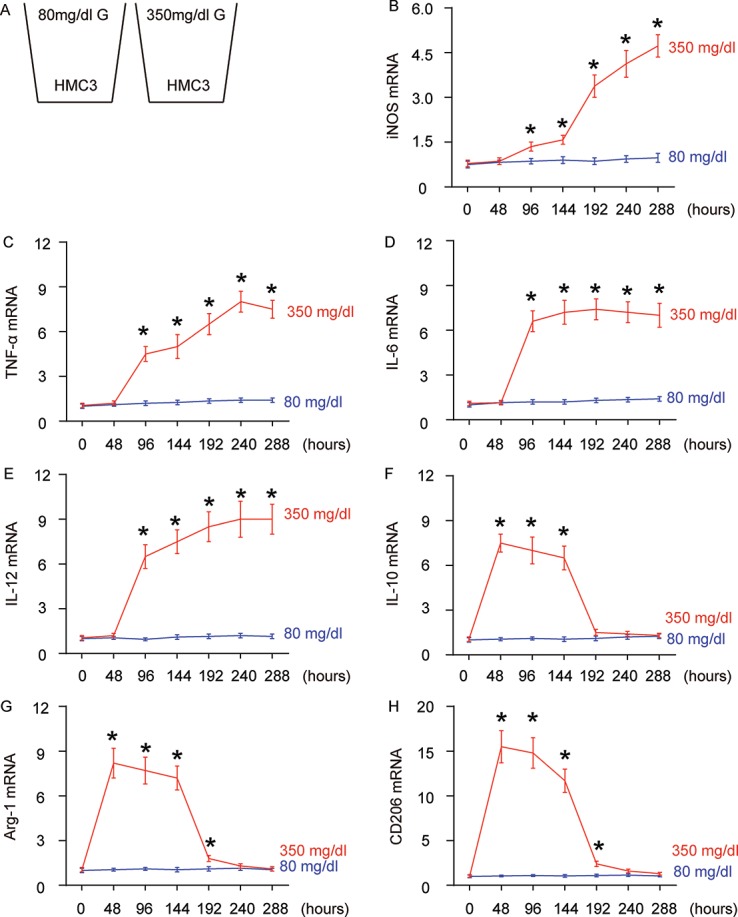
**High glucose alters microglia polarization with time.** (**A**) The microglia cell line HMC3 was cultured in a normal physiological glucose level (80 mg/dl G) and high glucose level (350 mg/dl G), respectively, for 288 hours. Some cells were harvested every 48 hours. (**B**–**G**) RT-qPCR for iNOS (**B**), TNF-α (**c**), IL-6 (**D**), IL-12 (**E**), IL-10 (**F**), Arg-1 (**G**) and CD206 (**H**) mRNA levels in HMC3 cells at different time points. N=5. *p<0.05.

Diabetic patients are prone to developing AD through undetermined molecular mechanisms. Given the important role of microglia and their polarization in aging and neurodegeneration, we hypothesized that high glucose (HG) may influence microglia differentiation and polarization, which subsequently affects the neurodegeneration process. In order to test this hypothesis, a microglia line HMC3 was cultured in normal physiological glucose (80 mg/dl; NG) or high glucose (350 mg/dl; HG), for 288 hours. This system allows examination of the direct effect of hyperglycemia (in diabetes) on microglia (Figure 1A).

We found that iNOS (Figure 1B) and TNF-α (Figure 1C) mRNA were not altered in 48 hours’ HG culture, but were significantly and continuously increased as early as 96 hours’ culture. The expression pattern of IL-6 (Figure 1D) and IL-12 (Figure 1E) mRNA levels was similar, but the further increases after 96 hours’ HG-culture were not obvious. In addition to these proinflammatory factors/cytokines, we also assessed IL-10, an anti-inflammatory cytokine in cultured cells. We found that levels of IL-10 were significantly and dramatically increased as early as in 48 hours’ HG-culture, were slightly and continuously decreased afterwards, and were nearly gone at 192 hours (Figure 1F). Two factors that are highly expressed in M2 macrophages/microglia, Arg-1 (Figure 1G) and CD206 (Figure 1H) had a similar expression pattern. To summarize the profile changes in HG-cultured microglia, activation of Arg-1, CD206 and IL-10 at 48 hours’ HG-culture suggested an M2 macrophage polarization. Moreover, lack of iNOS, TNF-α, IL-6 and IL-12 suggested that it was an M2a-like differentiation. At 96 hours and 144 hours, iNOS, TNF-α, IL-6 and IL-12 were all activated, while Arg-1, CD206 and IL-10 remained. Hence, the microglia appeared to polarize to a M2b subtype during this period. From 192 hours’ HG-culture, iNOS, TNF-α, IL-6 and IL-12 levels did not drop, while the expression of Arg-1, CD206 and IL-10 was nearly gone, suggesting an M1-like polarization. Together, chronic HG likely induces a progressive polarization of microglia, from M2a to M2b, and subsequently to M1.

### Concomitant alteration of ERK5 activation with polarization of HG-cultured microglia

**Figure 2:**
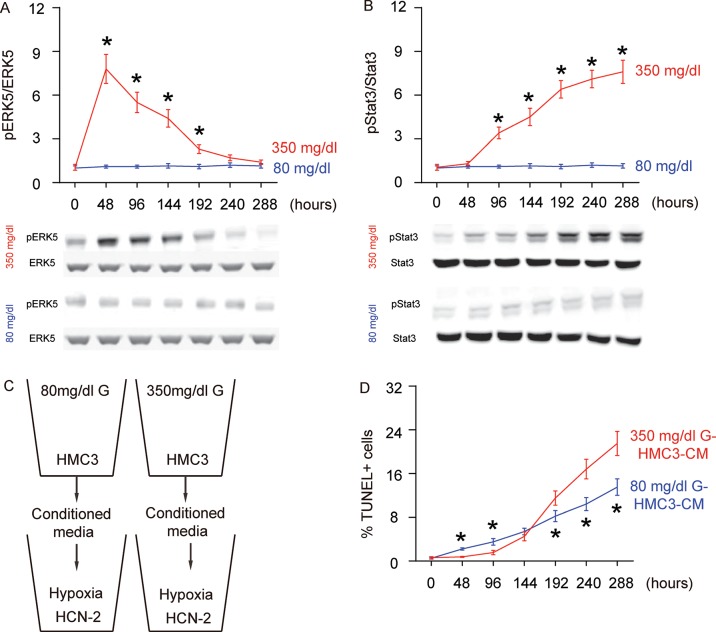
**Concomitant alteration of ERK5 activation with polarization of HG-cultured microglia, which affects the apoptosis of neuronal cells cultured in hypoxic conditions.** (**A**) Western blot for phosphorylated ERK5 (pERK5), compared to the total ERK5 levels in HG- or NG-cultured microglia in different time courses. (**B**) Western blot for phosphorylated Stat3 (pStat3), compared to the total ERK5 levels in HG- or NG-cultured microglia in different time courses. (**C**) Conditioned media (CM) from polarized microglia in different conditions and different time courses were added into a neuronal cell line, HCN-2, cultured in hypoxic conditions. (**D**) TUNEL assay on HCN-2 cells. N=5. *p<0.05.

ERK5 signaling is critical for neuroprotection and neurogenesis, although a role in microglia polarization is unknown. We examined the levels of phosphorylated ERK5 (pERK5), an active form of ERK5, compared to total ERK5 levels, in HG- or NG-cultured microglia in different time courses. While the ratio of pERK5 versus ERK5 remained unchanged in NG-cultured microglia, HG induced an immediate increase in pERK5/ERK5 as early as 48 hours. However, this increase in pERK5/ERK5 became compromised with time and was completely gone at 288 hours (Figure 2A). ERK5 activation has been shown to induce M2 macrophage polarization by suppressing Stat3 phosphorylation [15], which was thus tested in this model. Indeed, we found that while the ratio of pStat3 to Stat3 remained unchanged in NG-cultured microglia, HG induced an immediate and continuous increase in the pStat3/Stat3 ratio starting at 96 hours (Figure 2B). Thus, ERK5 activation by HG may affect microglia polarization through inhibition of Stat3 phosphorylation.

### Conditioned media from differently polarized microglia affect the apoptosis of neuronal cells cultured in hypoxia

To evaluate if the HG-polarized microglia may affect the development of AD, we isolated conditioned media (CM) from NG- and HG- cultured microglia at different time courses and added them into a neuronal cell line, HCN-2, which was cultured in hypoxic conditions (Figure 2C). We found that compared to HCN-2 cells cultured with CM from NG-cultured microglia, apoptosis in HCN-2 cells cultured with CM from HG-cultured microglia was significantly lower at 48 hours and 96 hours, similar at 144 hours, and significantly higher at the time courses later than 192 hours (Figure 2D). These data are consistent with an M2a-M2b-M1 microglia polarization process under long term HG culture.

### Suppressing ERK5 activation prevents HG-induced M2a polarization of microglia and induces earlier olarization of M2b-like followed by M1-like microglia

**Figure 3:**
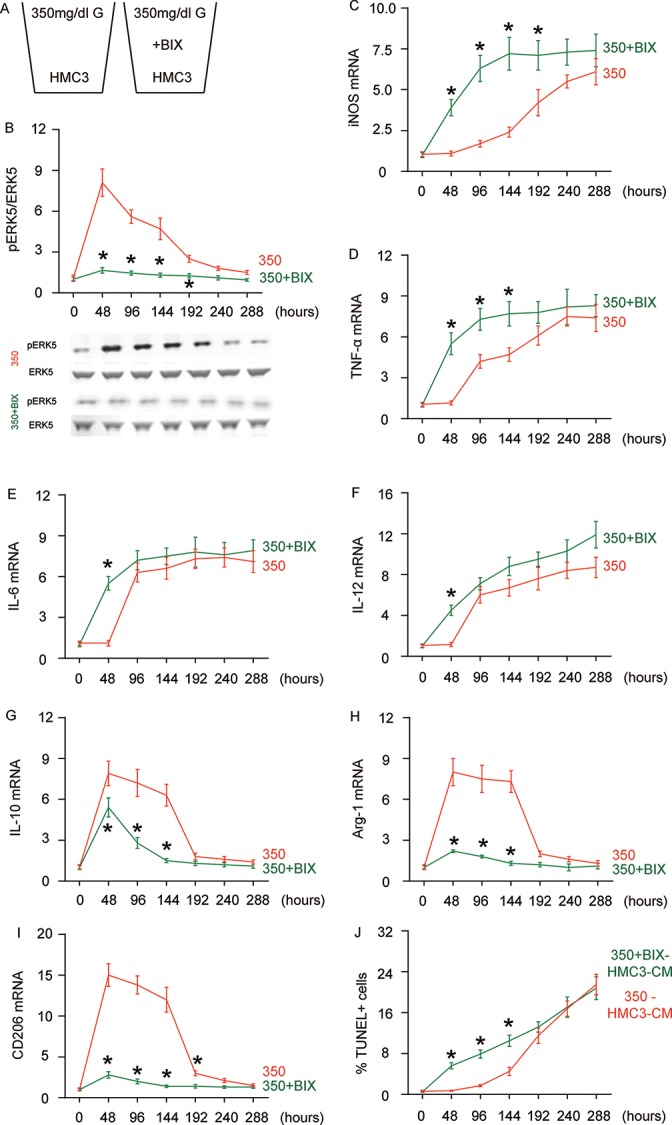
**Suppressing ERK5 activation prevents HG-induced M2a polarization of microglia and induces earlier polarization of M2b-like followed by M1-like microglia.** (**A**) BIX021895 (BIX) was applied to the HG-cultured microglia. (**B**) Western blot for pERK5, compared to the total ERK5 levels in HG- cultured microglia at different time courses, with or without BIX. (**C**–**I**) RT-qPCR for iNOS (**C**), TNF-α (**D**), IL-6 (**E**), IL-12 (**F**), IL-10 (**G**), Arg-1 (**H**) and CD206 (**I**) mRNA levels in HG- cultured microglia at different time points. (**J**) TUNEL assay on HCN-2 cells. N=5. *p<0.05.

In order to assess whether the HG-induced changes in ERK5 activation may be a crucial regulator for microglia polarization, we used BIX021895 (BIX), a specific inhibitor of ERK5 activation, in the HG-cultured microglia (Figure 3A). The effects of BIX on ERK5 phosphorylation were first confirmed (Figure 3B). We found that the presence of BIX induced early activation (as early as 48 hours) of iNOS (Figure 3C), TNF-α (Figure 3D), IL-6 (Figure 3E) and IL-12 (Figure 3F) activation. Conversely, the M2-associated factors IL-10 (Figure 3G), Arg-1 (Figure 3H) and CD206 (Figure 3I) exhibited significantly compromised activation or did not activate at all at 48 hours, 96 hours and 144 hours. To summarize the profile changes in BIX-treated, HG-cultured microglia, the M2a-like polarization was abolished at 48 hours. The M2b-like changes occurred likely as early as 48 hours and the microglia displayed M1-like changes earlier as well. The CM from HG- cultured microglia with or without BIX at different time courses were isolated and added into HCN-2 cells that were cultured in hypoxic conditions. We found that, compared to HCN-2 cells cultured with CM from HG-cultured microglia, apoptosis in HCN-2 cells cultured with CM from BIX-treated, HG-cultured microglia was significantly higher at 48 hours, 96 hours and 144 hours, but became similar at 192 hours and afterward (Figure 3J). This phenomenon likely resulted from the loss of M2a polarization at early culture, since M2a microglia protect neuronal cells in hypoxic culture. Together, these data suggest that suppressing ERK5 activation prevents HG-induced M2a polarization of microglia and induces earlier development of the M2b-like followed by M1-like phenotype.

### Sustained ERK5 activation maintains HG-induced M2a polarization of microglia

**Figure 4:**
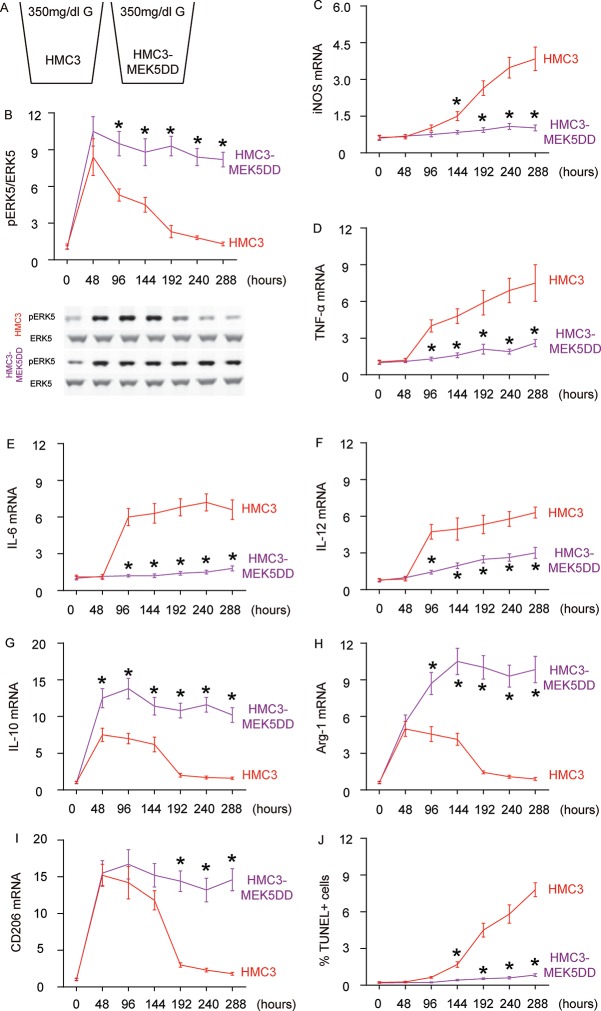
**Sustained ERK5 activation maintains HG-induced M2a polarization of microglia.** (**A**) HG-culturing of MEK5DD-transfected and control microglia. (**B**) Western blot for pERK5, compared to the total ERK5 levels in HG- cultured, MEK5DD-transfected and control microglia at different time courses. (**C**–**I**) RT-qPCR for iNOS (**C**), TNF-α (**D**), IL-6 (**E**), IL-12 (**F**), IL-10 (**G**), Arg-1 (**H**) and CD206 (**I**) mRNA levels in HG- cultured, MEK5DD-transfected and control microglia at different time points. (**J**) TUNEL assay on HCN-2 cells. N=5. *p<0.05

**Figure 5:**
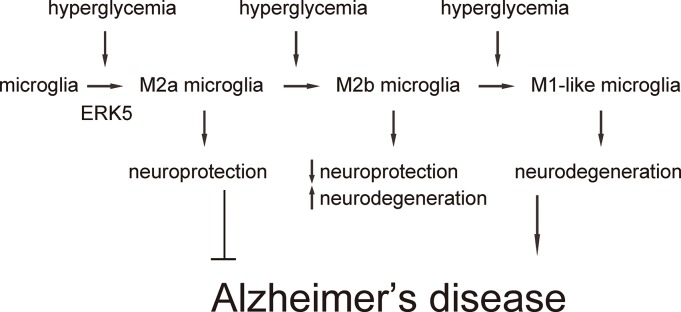
**Schematic of the model.** Our findings are summarized in a schematic, showing that chronic hyperglycemia may induce a gradual alteration of microglia polarization into an increasingly subtype, which could be suppressed by sustained activation of ERK5 signaling.

Next, we examined the effects of sustained ERK5 activation on HG-induced M2a polarization of microglia. For this aim, microglia were transfected with a sustained activated form of MEK5 (MEK5DD) or control plasmid (Figure 4A). The effects of MEK5DD on sustained ERK5 activation were first confirmed (Figure 4B). We found that sustained ERK5 activation by MEK5DD abolished the activation of iNOS (Figure 4C), TNF-α (Figure 4D), IL-6 (Figure 4E) and IL-12 (Figure 4F), and induced sustained activation of M2-associated factors IL-10 (Figure 4G), Arg-1 (Figure 4H) and CD206 (Figure 4I) to a greater extent. To summarize the profile changes in HG-cultured MEK5DD-transfected microglia, the M2a-like polarization was prolonged and the later differentiation into M2b/M1 phenotype was prevented. The CM from HG- cultured, MEK5DD-transfected or control microglia at different time courses were applied to HCN-2 cells that were cultured in hypoxic conditions. We found that, compared to HCN-2 cells cultured with CM from HG-cultured control microglia, apoptosis in HCN-2 cells cultured with CM from HG-cultured, MEK5DD-transfected microglia was significantly lower after 144 hours (Figure 4J). This phenomenon likely resulted from the maintenance of M2a polarization, which protects neuronal cells in hypoxic culture. Together, these data suggest that sustained ERK5 activation maintains HG-induced M2a polarization of microglia. Our findings are summarized in a schematic, showing that chronic hyperglycemia may induce a gradual alteration of microglia polarization into an increasingly proinflammatory subtype, which could be suppressed by sustained activation of ERK5 signaling (Figure 5). These findings provide an explanation for how the role of microglia in the development of AD is that of a “double-edged sword”.

## Discussion

ERK5 is the largest member of the mitogen-activated protein kinases (MAPK) family. Once stimulated, ERK5 is activated by the upstream kinase MEK5 [16]. In our previous study, we demonstrated robust activation of ERK5 in islets from pregnant mice [14]. Suppression of ERK5 activation significantly reduced beta cell proliferation in the gestational period, resulting in glucose intolerance and even gestational diabetes [14]. In the brain, ERK5 signaling has been shown to mediate neuroprotection against oxidation [17,18], and to regulate neurogenesis [18–23]. Interestingly, ERK5 activation has been shown to induce M2 macrophage polarization through suppression of Stat3 phosphorylation [15], which suggests a possible role for ERK5 in microglia polarization. Since microglia polarization has been associated with chronic inflammation in the brain, we hypothesized that hyperglycemia in diabetes may affect neurodegeneration by altering inflammatory status through ERK5-dependent microglia polarization. In the current study, with the help of a loss of function experiment using BIX and a gain of function experiment using MEK5DD-expressing microglia cells, we demonstrate a robust role for ERK5 signaling in the HG-mediated regulation of microglia polarization. Since we applied a simplified in vitro model in which the niche effects from the inflammatory brain were dismissed, the independent role of HG on microglia polarization and on neuronal cell aging and degeneration was evaluated in the absence of the effects of HG on neuronal cells. It is noteworthy that in the real progression of the disease, HG affects not only microglia, but also neuronal cells, endothelial cells, and mesenchymal cells, thereby contributing to the pathological effects of AD.

As mentioned earlier, microglia polarize to a spectrum of diverse subtypes with distinct physiologic and pathologic roles. Here, we provided evidence that HG first induced M2a-like polarization, which should have a protective effect on nearby neuronal cells. With time, this M2a-like polarization changes into M2b, a subtype characterized by production of some M1-proinflammatory factors concomitant with compromised M2-factors. This phenotype is likely an intermediate polarized subtype between M2a and M1. Indeed, at later stages, the polarized microglia appeared to be M1-like. It is highly possible that this change in microglia polarization is dynamic and continuous. Since we used a microglia cell line in this study, the effects of HG were likely rather uniform on individual cells. In other words, the cultured microglia likely respond to HG in a more synchronized way, compared to primary microglia.

To the best of our knowledge, our study is the first to address a direct effect of HG on microglia polarization, which contributes to an understanding of the pathogenesis of AD. Future experiments may be conducted to compare the effects of HG on microglia and bone marrow derived monocytes/macrophages in the brain, in order to distinguish their different roles in disease progression.

## Materials and Methods

### Experimental protocols and reagents

All animal experiments were approved by the Animal Research and Care Committee at Wenzhou Medical University. D-glucose was purchased from Sigma-Aldrich (Beijing, China) and applied to cultured cells at a concentration of either 80 mg/dl or 350 mg/dl glucose. The ERK5 inhibitor BIX02189 (Selleck Chemicals, Houston, TX, USA) was applied to the cultured cells at a dose of 5mM. The control is saline of the same volume.

### Cell culture and transfection

HMC3 (CRL-3304) is a human microglia cell line purchased from ATCC (ATCC, Rockville, MD, USA). HCN-2 (CRL-10742, ATCC) is a human cortical neuronal cell line. HMC3 cells were cultured in Eagle’s Minimum Essential Medium (EMEM, ATCC), 10% fetal bovine serum (FBS, ATCC) and certain concentration of D-glucose. HCN-2 cells were cultured in Dulbecco’s modified Eagle’s medium (DMEM, Invitrogen, Shanghai, China) with 4 mM L-glutamine adjusted to contain 1.5 g/L sodium bicarbonate, 10% FBS and 80 mg/dl D-glucose. Cells were maintained in a humidified chamber with 5% CO_2_ at 37 °C. The media were replaced every 3 days. Transfection of HMC3 was conducted with a pcDNA3-MEK5DD-HA (Catalog number: 65247; Addgene, Watertown, MA, USA) or control plasmid, using lipofectamine 3000 (Thermo Fisher Scientific, Inc., Waltham, MA, USA) [24].

### Quantitative PCR (RT-qPCR)

Total RNA was extracted using the RNeasy mini kit (Qiagen, Beijing, China), followed by reverse transcribing into complementary DNA (cDNA) with the RT Kit (Qiagen). RT-qPCR was performed in triplicates with the SYBR Green PCR Kit (Qiagen). All primers were purchased from Qiagen. Values of genes were determined by sequential normalization to the housekeeping gene α-tubulin and the experimental controls.

### Western blot

As described previously [14,25,26], the cultured cells were homogenized in protein lysis buffer (Bio-rad, Beijing, China), followed by protein concentration assessment with a BCA protein assay kit (R&D systems, Beijing, China). Western blot was performed with the following primary antibodies: rabbit anti-ERK5 (Cell signaling, San Jose, CA, USA), rabbit anti-pERK5 (Cell signaling), rabbit-anti-Stat3 (Cell Signaling) and rabbit anti-pStat3 (Cell Signaling). The secondary antibody was HRP-conjugated anti-rabbit (DAKO, Beijing, China). Image acquisition and densitometric analysis of the gels were performed with NIH ImageJ software (Bethesda, MA, USA).

### TUNEL assay

TUNEL assay was done as previously described [26], with a specific detection kit (Roche Applied Science, Nutley, NJ, USA). The number of TUNEL+ cells divided by total cells constituted the percentage of apoptotic cells.

### Statistical analysis

Statistical analysis was performed using GraphPad Prism 7 (GraphPad Software, San Diego, CA, USA). Analysis was conducted by one-way ANOVA with a Bonferroni correction, followed by Fisher’s Exact Test upon necessity. All values are depicted as mean ± standard deviation from 5 repeats and are considered significant if p < 0.05.
